# Supplementary material for: Skill retention with ultrasound curricula
Source: PLoS One. 2020 Dec 3;15(12):e0243086. doi: 10.1371/journal.pone.0243086 (PMC7714199; doi:10.1371/journal.pone.0243086)
Supplement: S1 Fig — (DOCX) [file pone.0243086.s001.docx]

**S1 Fig**

**Name ____________________________________ Date _________________ PGY Level _______**

**Please check one of the following as appropriate:**

Pre-training assessment____ Immediate post-training assessment____

6-month follow-up____ 12-month follow-up____

**Thoracic Ultrasound Knowledge and Skills Assessment**

For the following questions in parts 1-3, please refer to this case vignette:

Karl is a 60-year-old male non-smoker with a past medical history significant only for diastolic heart failure and asthma who presents with acute onset of dyspnea that started last night. His dyspnea is worsened by exertion and with deep breaths. He had subjective fevers but has not checked his temperature; he has had no chills. He has been adherent with his medications at home including diuretics as well LABA/ ICS inhaler. His exam is notable for mild tachypnea, mildly diminished air exchange, chronic lower extremity edema. Patient’s pulmonary function tests from 3 months ago showed airflow limitation with significant post-bronchodilator reversibility.

**Part 1: Image acquisition and identification of anatomy**

Please perform a thoracic ultrasound to help you refine your differential diagnosis.

Patient 1:

1. Content:
   1. Correctly identifies muscle wall (no = 0 pt, yes = 1 pt)
   2. Correctly identifies pleural line (no = 0, yes = 1pt)
   3. Correctly identifies pleural sliding (no = 0, yes = 1pt)
   4. Correctly identifies rib shadows (no = 0 pt, yes = 1 pt)
   5. Correctly identifies muscle wall n M mode (no = 0 pt, yes = 1 pt)
   6. Correctly identifies pleural line in M mode (no = 0, yes = 1pt)
   7. Correctly identifies pleural sliding in M mode (no = 0, yes = 1pt)
   8. Correctly identifies a-lines (no = 0, yes= 1 pt)
   9. Correctly identifies b-lines if present (no = 0, yes= 1 pt)
   10. Correctly identifies z-lines (no = 0, yes = 1 pt)
   11. Correctly identifies lung pulse if present (no = 0, yes = 1pt)
   12. Correctly identifies presence or absence of lung point (no = 0, yes =1 pt)
   13. Correctly identifies diaphragm(no = 0 pt, yes = 1 pt)
   14. Correctly identifies liver (no = 0 pt, yes = 1 pt)
   15. Correctly identifies spleen (no = 0 pt, yes = 1 pt)
   16. Correctly identifies caudad vs cephalad (no= 0, yes = 1pt)
2. Examine enough sites to rule out pneumothorax and pleural effusion on each side of the thorax.
   1. Evaluates 4 sites on each side for lung sliding (no = 0, yes = 1 pt)
   2. Evaluated for pleural effusion posteriorly bilaterally (no = 0, yes = 1 pt)
3. Image optimization: Adjust image as necessary to adequately assess pleura/ lungs (e.g. gain, depth, positioning)
   1. Adjusts gain (no = 0, yes = 1 pt)
   2. Adjusts depth (no = 0, yes= 1 pt)
   3. Orient probe marker correctly (no=0, yes = 1pt)
   4. Operator faces machine (no = 0, yes = 1 pt)
   5. Correct transducer setting is chosen on ultrasound machine (no = 0, yes =1 pt)

**Name ____________________________________ Date _________________ PGY Level _______**

**Please check one of the following as appropriate:**

Pre-training assessment____ Immediate post-training assessment____

6-month follow-up____ 12-month follow-up____

**Part 2 Image interpretation:**  Each correct answer is worth 1 pt.

Images 1-6 reflect possible findings on thoracic ultrasound for this patient. **A video/GIF will be shown for each question below:**

1. If this is what you saw on Karl’s ultrasound, which diagnosis would be most consistent with your findings?
   1. Decompensated heart failure
   2. Pneumothorax
   3. No pathology identified
   4. Pleural effusion
2. If this is what you saw on Karl’s ultrasounds, which diagnosis would be most consistent with your findings?
   1. Decompensated heart failure
   2. Pneumothorax
   3. No pathology identified
   4. Pleural effusion
   5. Upper respiratory tract infection
3. If this is what you saw on Karl’s ultrasound, is it safe to drain a pleural effusion?
   1. Yes
   2. No
4. If this is what you saw on Karl’s ultrasound, which diagnosis would be most consistent with your findings?
   1. Upper respiratory tract infection
   2. Pulmonary edema
   3. Asthma exacerbation
   4. Pneumothorax
5. If this is what you saw on Karl’s ultrasound, which diagnosis would be most consistent with your findings?
   1. Pneumothorax
   2. Pulmonary edema
   3. Pleural effusion
   4. Asthma exacerbation
6. If this is what you saw on Karl’s ultrasound, which diagnosis would be most consistent with your findings?
   1. Pulmonary edema
   2. Asthma exacerbation
   3. Pulmonary embolism
   4. Complex pleural effusion
   5. Upper respiratory tract infection
7. If you saw this on Karl’s ultrasound, this would be an indication of:
   1. Pulmonary edema
   2. Probable normal variant
   3. Interstitial pneumonia
   4. Pulmonary fibrosis
8. This image can be seen with which of the following:
   1. Pneumonia
   2. Emphysema
   3. Normal Lung
   4. A & B

**Name ____________________________________ Date _________________ PGY Level _______**

**Please check one of the following as appropriate:**

Pre-training assessment____ Immediate post-training assessment____

6-month follow-up____ 12-month follow-up____

**Part 3 : Management**

1. If this is what you saw on Karl’s ultrasound, what would be your next step?
   1. Decompress Pneumothorax
   2. Treat for asthma exacerbation with steroids and nebulizer
   3. Give antibiotics
   4. Give diuretics
2. If this is what you saw on Karl’s ultrasound, after placement of subclavian line, what would be your next step?
   1. Decompress Pneumothorax
   2. Order stat Computerized Tomography (CT) Scan Chest Non-Contrast
   3. Order stat supine radiograph
   4. No intervention
3. If this is what you saw on Karl’s ultrasound, what would be your next step?
   1. Decompress pneumothorax
   2. Treat asthma exacerbation
   3. Order chest radiograph
   4. Treat decompensated congestive heart failure
4. If this is what you saw on Karl’s ultrasound, what would be your next step?
   1. Treat for chronic obstructive pulmonary disease exacerbation
   2. Decompress pneumothorax
   3. Order chest radiograph
   4. Perform thoracentesis
5. If this is what you saw on Karl’s ultrasound, what would be your next step?
   1. Diuresis
   2. Decompress pneumothorax
   3. Administer antibiotics
   4. Perform thoracentesis

**Part 4 Knowledge assessment**

For this part, do not refer to the clinical vignette. These are knowledge-based questions unrelated to the clinical vignette above.

1. Which of the following rules out a pneumothorax in the lung field you are examining with ultrasound?
   1. Lung sliding
   2. Lung pulse
   3. Lung point
   4. B-lines
   5. a, b or d

1. Multiple bilateral diffuse B-lines are possibly consistent with the following pathologic processes:
   1. Pulmonary edema
   2. Pneumothorax
   3. Interstitial pneumonia
   4. Pulmonary fibrosis
   5. a, c, or d
