# Supplementary material for: Skill retention with ultrasound curricula
Source: PLoS One. 2020 Dec 3;15(12):e0243086. doi: 10.1371/journal.pone.0243086 (PMC7714199; doi:10.1371/journal.pone.0243086)
Supplement: S1 Table — (DOCX) [file pone.0243086.s002.docx]

S1 Table

| **1 point for each correct answer** | **Pre-training** | **Pre-training** | **Immediate post-training** | **Immediate post-training** | **6-month follow-up** | **6-month follow-up** | **12-month follow-up** | **12-month follow-up** |
| --- | --- | --- | --- | --- | --- | --- | --- | --- |
| **Question** | **Intervention group N=14** | **Control group N=14** | **Intervention group N=14** | **Control group N=14** | **Intervention group N=14** | **Control group N=12** | **Intervention group N=14** | **Control group N=12** |
| **Image interpretation:** **A video/GIF was provided for each image interpretation** | Correct responses n(%) | Correct responses n(%) | Correct responses n(%) | Correct responses n(%) | Correct responses n(%) | Correct responses n(%) | Correct responses n(%) | Correct responses n(%) |
| 1 | 5 (36%) | 7 (50%) | 9 (64%) | 5 (36%) | 10 (71%) | 6 (50%) | 12 (86%) | 7 (58%) |
| 2 | 5 (36%) | 6 (43%) | 10 (71%) | 10 (71%) | 7 (50%) | 9 (75%) | 13 (93%) | 11 (92%) |
| 3 | 11 (78%) | 14 (100%) | 14 (14%) | 14 (100%) | 14 (100%) | 11 (92%) | 12 (86%) | 12 (100%) |
| 4 | 8 (57%) | 9 (64%) | 13 (93%) | 13 (93%) | 13 (93%) | 12 (110%) | 14 (100%) | 12 (100%) |
| 5 | 11 (78%) | 8 (57%) | 11 (78%) | 12 (86%) | 14 (100%) | 12 (100%) | 14 (100%) | 12 (100%) |
| 6 | 14 (100%) | 12 (86%) | 14 (100%) | 14 (100%) | 14 (100%) | 12 (100%) | 14 (100%) | 12 (100%) |
| 7 | 6 (43%) | 8 (57%) | 12 (86%) | 9 (64%) | 9 (64%) | 8 (67%) | 12 (86%) | 8 (67%) |
| 8 | 4 (28%) | 11 (78%) | 6 (43%) | 7 (50%) | 4 (28%) | 6 (50%) | 4 (28%) | 5 (42%) |
| **Management** |  |  |  |  |  |  |  |  |
| 1 | 8 (57%) | 6 (43%) | 8 (57%) | 11 (78%) | 12 (86%) | 12 (100%) | 14 (14%) | 11 (92%) |
| 2 | 6 (43%) | 3 (21%) | 8 (57%) | 5 (36%) | 11 (78%) | 2 (17%) | 12 (86%) | 9 (75%) |
| 3 | 9 (64%) | 7 (50%) | 12 (86%) | 12 (86%) | 11 (78%) | 10 (83%) | 10 (71%) | 10 (83) |
| 4 | 10 (71%) | 13 (93%) | 12 (86%) | 13 (93%) | 14 (100%) | 12 (100%) | 14 (14%) | 12 (100%) |
| 5 | 10 (71%) | 9 (64%) | 11 (78%) | 10 (71%) | 12 (86%) | 7 (58%) | 12 (86%) | 10 (83%) |
| **Knowledge assessment** |  |  |  |  |  |  |  |  |
| 1 | 5 (36%) | 3 (21%) | 13 (93%) | 10 (71%) | 10 (71%) | 7 (58%) | 12 (86%) | 9 (75%) |
| 2 | 5 (36%) | 11 (78%) | 11 (78%) | 11 (78%) | 9 (64%) | 8 (67%) | 8 (57%)) | 7 (58%) |
