# Supplementary material for: Skill retention with ultrasound curricula
Source: PLoS One. 2020 Dec 3;15(12):e0243086. doi: 10.1371/journal.pone.0243086 (PMC7714199; doi:10.1371/journal.pone.0243086)
Supplement: Attachment — Submitted filename: Response to Reviewers R2 10.21.2020.docx [file pone.0243086.s004.docx]

Response to Reviewers

Reviewer #2: On re-review with the changes incorporated the Authors have made clear the design, findings and limitations and have better incorporated existing literature for comparison. My only comments now are stylistic:

Minor suggestions

Line 29, 74: ”valuable, but optimal…”

Line 32: from baseline instead of after baseline

Line 34: This sentence reads awkwardly

Line 80: How does this tie to skill retention

Line 194: I might say “An alternative yearlong lecture based model , even with personal HUD, showed no improvement in knowledge or image interpretation score.”

**Authors’ response:** Thank you for your kind remarks, detailed review of the revision and stylistic suggestions. We have incorporated all of the suggestions in the revised manuscript.

Reviewer #3: The authors have addressed my concerns. However two issues remain.

A major limitation is that it is unknown how many scans both groups performed during the study besides the study protcol. This study supports that proctored scanning helps, but the amount of the effect in unknown. If control group made no scans than 20 additonal scan is useful in increasing skills. If both groups made an additional 30 ultrasound, the most important explanation is that supervized scanning with bedside feedback is most important, not the amount.

**Authors’ response:** We appreciate the reviewers concerns and have previously included this limitation in our previous manuscript. We did not consider tracking non-study related POCUS exams in either group as part of the protocol. Based on previous literature in this area, we also noted that Mellor et al, did not capture scans performed in non-study related activities and Kelm et al did not account for ultrasound exposure during the follow-up period, listing this as a limitation. However, we do appreciate the reviewers concerns and will consider this limitation in our future work in this area.

it is still unclear to me how the practical assessment scores are computed. The supplement shows 23 points to be earned, but scores in tables are up to 86?

This needs to be explainedto be able to interpret the conclusions

**Authors’ response:** The scores are reported as percentages, range 0-100% based on the number correct responses/the number of questions. We included a sentence in the abstract and the methods section of the paper to add clarification. *Scores are reported as a percentage of the number of correct responses/number of questions (range 0 - 100%).*
